# Hepatitis B Virus Reactivation with Immunosuppression: A Hidden Threat?

**DOI:** 10.3390/jcm13020393

**Published:** 2024-01-11

**Authors:** Sama Anvari, Keith Tsoi

**Affiliations:** 1 Division of Gastroenterology, Department of Internal Medicine, McMaster University, Hamilton, ON L8S 4L8, Canada; sama.anvari@medportal.ca; 2 Division of Gastroenterology, St. Joseph’s Healthcare Hamilton, Hamilton, ON L8N 4A6, Canada

**Keywords:** hepatitis B virus, reactivation, immunosuppression, hepatitis

## Abstract

Hepatitis B virus (HBV) reactivation in the setting of immunosuppressive therapy is an increasingly recognized and preventable cause of elevated liver enzymes and clinical hepatitis in treated patients. However, not all immunosuppressive therapies confer the same risk. The purpose of this article was to review the literature on risks of HBV reactivation associated with immunosuppressive agents and propose a management algorithm. We searched Google Scholar, PubMed, and MEDLINE for studies related to hepatitis B reactivation and various immunosuppressive agents. The risk of HBV reactivation was found to differ by agent and depending on whether a patient had chronic HBV (HBsAg+) or past HBV (HBsAg−, anti-HBc+). The highest risk of reactivation (>10%) was associated with anti-CD20 agents and hematopoietic stem cell transplants. Multiple societies recommend HBV-specific anti-viral prophylaxis for patients with positive HBsAg prior to the initiation of immunosuppressive therapy, while the guidance for HBsAg− patients is more variable. Clinicians should check HBV status prior to beginning an immune-suppressive therapy. Patients with positive HBsAg should be initiated on antiviral prophylaxis in the majority of cases, whereas HBsAg− individuals should be evaluated on a case-by-case basis. Further research is required to determine the optimum duration of therapy.

## 1. Introduction

Hepatitis B virus (HBV) is a global health concern, with an estimated 2 billion individuals worldwide encountering the virus during their lifetime [1]; of these, approximately 250 million develop chronic HBV infection (positive HBsAg). The World Health Organization (WHO) proposed a strategy to eliminate viral hepatitis as a major public health threat by 2030, aiming to target HBV spread and morbidity through the prevention of vertical transmission, widespread vaccination, and the use of vaccination and antiviral treatment for individuals with chronic HBV [2]. Some progress has been made, with improved HBV vaccination rates and reductions in global infections noted between 2015 and 2020 [3]. Despite this, a 2023 modeling study estimated the global prevalence of HBV infection at 3.2%, and global rates of diagnosis and treatment remain well below estimated targets [4,5]. Major challenges in the elimination of HBV as a public health threat include high levels of endemicity in low- and middle-income countries and a need for improved infrastructure to reduce transmission and broaden access to care. Recent research efforts have also focused on devising a safe and effective cure for HBV, though this will likely require a combination of multiple agents to be successful. Though agents such as bepisovirsen are currently being investigated in early phase 2 and 3 clinical trials [6,7,8], a functional cure has remained elusive due to the nature of HBV infection.

HBV is a hepatotropic virus that may be transmitted via percutaneous, perinatal, or sexual exposures. Upon the infiltration of host hepatocytes, the virus’s double-stranded DNA (dsDNA) enters the nucleus, whereafter a viral polymerase repairs the dsDNA into full-length, covalently closed circular DNA (cccDNA). This cccDNA acts as a template for viral replication and may persist for decades despite host clearance of an acute HBV infection and associated serologic resolution (i.e., the clearance of HBsAg and development of anti-HBc) [9]. Although there have been significant advances in the treatment of HBV that have allowed individuals to attain a functional cure (the loss of HBsAg, undetectable HBV DNA, and normalized liver enzymes), a complete cure (the eradication of all HBV genomic material, including cccDNA and integrated HBV DNA sequences) for HBV has not yet been achieved [5]. Persistent cccDNA can, therefore, serve as a potential reservoir for future viral replication if immune control of the virus is affected.

**Figure 1:**
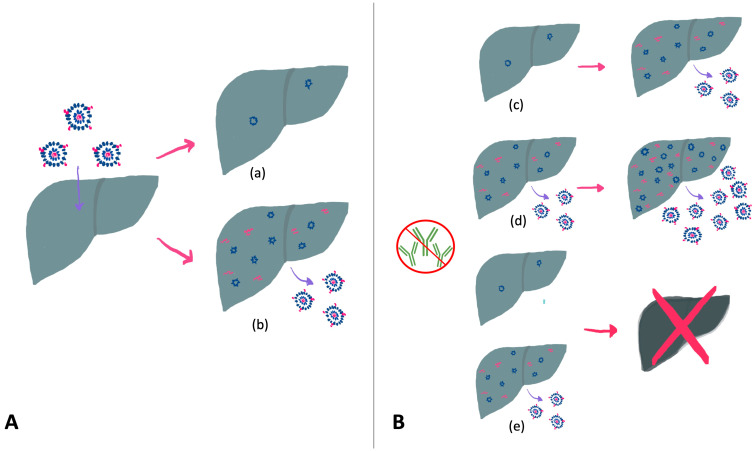
(**A**) Illustration of potential outcomes following exposure to the HBV virus. Individuals may spontaneously clear the virus, though cccDNA persists in host hepatocytes (HBsAg−, anti-HBc+) (a) or may develop chronic HBV infection (HBsAg+) (b). (**B**) Potential outcomes of HBV reactivation with immunosuppression, which may include seroconversion and viral replication in HBsAg− individuals (c), increased viral replication and hepatitis (d), or progression to acute liver failure (e). Adapted from [9].

HBV reactivation is defined in the 2018 AASLD guidelines as “the loss of HBV immune control in HBsAg-positive, anti-HBc-positive or HBsAg-negative, anti-HBc-positive patients receiving immunosuppressive therapy for a concomitant medical condition” [10] and reflects the host immune system’s inability to control the infection. HBV reactivation, loosely defined as increased viral replication or reverse seroconversion, may occur spontaneously [11,12] but is more commonly triggered by immunosuppressive therapies due to a loss of immune control (particularly adaptive T and B cell responses) [13]. Figure 1 illustrates this process. Advances in treatment paradigms for malignant and immune-mediated conditions have led to the development and widespread use of these agents. At the same time, there has been an increased recognition of HBV reactivation as a cause of elevated liver enzymes and clinical hepatitis in patients receiving immunosuppressive therapy [14]. This phenomenon was first appreciated in the context of chemotherapy for hematologic malignancies [15,16] but has since been described with the use of a variety of other agents. HBV reactivation may result in acute hepatitis and, rarely, death from fulminant hepatic failure. Thankfully, it is preventable with the advent of potent HBV-specific antiviral therapies. Many comprehensive reviews have described the risk of HBV reactivation associated with specific therapies. The purpose of this review is to summarize the literature regarding the risk of reactivation associated with different classes of immunosuppressive agents and discuss the suggested monitoring and management of patients at risk for HBV reactivation.

## 2. Sources and Selection Criteria

This was a narrative review. We comprehensively searched Google Scholar, PubMed, and MEDLINE for studies published from database inception to November 2023, using the following search terms in combination: “HBV”, “Hepatitis B” and “chronic B hepatitis” with “reactivation”, “flare”, “immunosuppression”, “liver failure” and, where applicable, the name of the immunosuppressive agent in question. References of published studies were also searched manually to ensure relevant studies were not missed. We prioritized the citation of evidence-based guidelines, systematic reviews (and their meta-analyses, where available), and high-quality prospective studies when available. However, perspectives from high-quality review articles and case series were also included. Studies were excluded if they were conducted in animal models or were unrelated to HBV reactivation in the context of immunosuppression.

## 3. Hepatitis B Reactivation: An Overview

### 3.1. Definitions

Individuals at risk for HBV reactivation fall into two main categories: those positive for hepatitis B surface antigen (HBsAg+), and those negative for HBsAg (HBsAg−) and positive for hepatitis B core antibody (anti-HBc). Individuals who are HBsAg− who also have a positive hepatitis B surface antibody (anti-HBs) may be described as having resolved HBV [17], though the risks of reactivation in this population are less clear and will be discussed later. HBV reactivation presents clinically as an increase in HBV DNA compared to baseline and/or a potential reverse seroconversion from HBsAg− to HBsAg+ in individuals with positive anti-HBc, with sequelae of hepatitis in some cases [10].

Various specific virologic and serologic criteria for HBV reactivation have been used in individual studies. However, a recent systematic review and meta-analysis proposed the following definitions upon a review and synthesis of the available literature and guidelines, which will be used for the purpose of this review [18]. For patients with positive HBsAg, HBV reactivation was defined as a ≥2 log (100-fold) increase in HBV DNA compared to baseline, an HBV DNA level of ≥1000 IU/mL if HBV DNA was previously detectable, or an HBV DNA level of ≥10,000 IU/mL if a baseline level is not available. For individuals with negative HBsAg and positive anti-HBc, reactivation was defined as seroconversion from HBsAg− to HBsAg+, the new detection of quantifiable HBV DNA if previously undetectable, HBV DNA ≥ 100 IU/mL if previously unknown or detected but not quantifiable, or a ≥1 log (10-fold) increase in HBV DNA if previously quantifiable. This definition is in keeping with most major society definitions, though the specific cutoffs vary slightly [19]. HBV-reactivation-associated hepatitis is broadly defined as an increase in ALT to ≥3× the baseline (if baseline ALT is elevated) or the ULN (if baseline ALT is normal) in the absence of other causes. “Severe” hepatitis was defined as a baseline ALT ≥10× ULN/baseline. Finally, HBV-associated liver failure is defined as one of impaired synthetic function (total bilirubin > 51.3 μmol/L or international normalized ratio > 1.5), ascites, encephalopathy, or death following HBV-associated liver failure attributed to HBV reactivation [10].

### 3.2. Natural History and Risk Factors for HBV Reactivation

**Figure 2:**
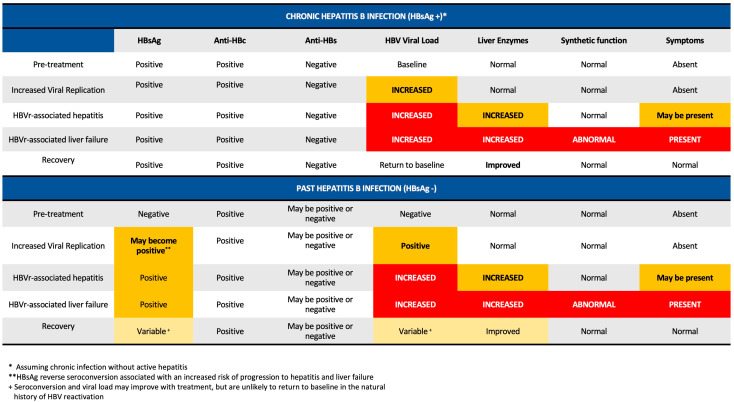
Graphic illustration of expected serology and laboratory values in the stages of HBV reactivation. Adapted from [20].

HBV reactivation often occurs in three phases: an increase in viral replication, the appearance of disease activity, and either subsequent recovery or progression to liver failure and, rarely, death [20]. This process is illustrated in Figure 1B and Figure 2. In the initial phase, exposure to immunosuppression may result in increased viral replication and, in the case of HBsAg− individuals, reverse seroconversion. In this stage, patients may be relatively asymptomatic, and their liver enzymes can remain normal. The timing of onset of HBV reactivation can vary from within weeks of initiating therapy to a year after treatment cessation [20]. A proportion of individuals may then progress to HBV-reactivation associated hepatitis, with increases in serum AST and ALT and, depending on severity, clinical symptoms of jaundice or right-upper-quadrant abdominal pain. This stage may occur days to weeks after an increase in HBV DNA. From this point, individuals may spontaneously recover (often in the context of completing therapy) or, in a small minority of patients, progress to acute liver failure. Some individuals may also experience a transient increase in HBV viral load without clinical consequences. Patients with previously negative HBsAg who experience reactivation may seroconvert with adequate therapy.

HBV reactivation depends on a variety of host, virologic, and treatment factors. Host factors associated with an increased risk of HBV reactivation include older age, the presence of cirrhosis, male sex, as well as the type of disease requiring immunosuppression (with lymphoma having the highest associated risk) [21,22,23]. Patients with positive HBsAg are at a 5–8-fold higher risk of reactivation than those with past or resolved HBV [21]. One systematic review of patients receiving chemotherapy found that patients who were HBsAg− had a reactivation rate of 14%, whereas those with resolved HBV had a reactivation rate of approximately 7.5% [17]. Of patients with positive HBsAg, a higher baseline viral DNA value and positive e antigen have been associated with a higher risk of reactivation, with the current AASLD guidelines recommending universal prophylaxis before any immunosuppressive or cytotoxic therapy in these individuals [10]. Some studies have also suggested that a non-A genotype, which represents approximately 83% of cases and is most prevalent among individuals from North Africa, Asia, most of South America, and southern Europe, may also increase risk [20,24]. Interestingly, there has been some evidence that the presence and titer of anti-HBs antibodies may be associated with some degree of protection against HBV reactivation [17], though data supporting this are limited. Finally, the type of immunosuppression given is a major risk factor for HBV reactivation; this will be discussed elsewhere.

Due to variability in definitions across studies and the presence of confounders, the incidence of morbidity and mortality associated with HBV reactivation has not been well established. Without treatment, rates of HBV reactivation and associated mortality may be as high as 40% with some therapies (such as B-cell-depleting therapies like rituximab and ofatumumab) [25,26,27]. Another systematic review estimated rates of liver failure among HBsAg-positive patients receiving chemotherapy to be up to 13.9% [28]. However, the true burden of HBV reactivation remains unclear and likely varies between patient populations and disease states.

## 4. Immunosuppressive Agents

### 4.1. Risk Stratification Overview

Immunosuppressive agents operate through a variety of different mechanisms and, as such, convey different baseline risks of reactivation. Societies such as the AASLD, as well as a recent systematic review and meta-analysis, have attempted to categorize the risk of HBV reactivation in order to determine whether preventive therapy is warranted prior to the initiation of a particular therapy, as well as how long it should be continued. The risk of HBV reactivation also varies based on the underlying HBV serologic status. These features are summarized in Table 1 [10,18,20,21].

### 4.2. Oncology

#### 4.2.1. Anti-CD20/B-Cell-Depleting Agents

B-cell-depleting agents such as rituximab are associated with the highest risk of HBV reactivation, as well as high mortality associated with HBV-related liver failure in both HBsAg+ and HBsAg− patients [29]. This is potentially related to both the properties of the therapy itself and the fact that patient populations treated with rituximab often have other underlying risk factors (e.g., hematologic malignancy) for HBV reactivation. One meta-analysis showed that lymphoma patients receiving rituximab had a significantly higher risk of HBVr compared to those who did not receive the drug as part of their regimen (10% vs. 4%), and anti-HBV prophylaxis is universally recommended in this group [17]. Though there is some variation in grading the risk of reactivation in HBsAg− patients as high versus moderate, all patients receiving anti-CD20 therapy who have been exposed to the HBV virus, regardless of anti-HBs status, are recommended to receive anti-HBV prophylaxis. Given the persistent immunosuppressive effects of anti-CD20 agents, these patients often require a prolonged course of prophylaxis, though there is no consensus on the exact duration—the European Association for Study of the Liver (EASL) recommends 18 months of prophylaxis [30], whereas the American Society of Clinical Oncology (ASCO) and the American Gastroenterological Association (AGA) suggest a shorter course of 6–12 months [25,31].

#### 4.2.2. Hematopoietic Stem Cell Transplant (HSCT)

Patients who undergo stem cell transplantation are treated with chemotherapy to ablate the recipient bone marrow, resulting in extreme immunosuppression; some studies have reported up to a 100% risk of reactivation and a 15% rate of liver failure in patients with positive HBsAg who do not receive prophylaxis [32,33]. This risk is also high in HBsAg− patients, with some data showing a 6–29% risk of reactivation in patients who underwent HSCT without prophylaxis [21,32]. Patients who received rituximab as part of their transplant regimen were found to be at a significantly higher risk, as were those who received a longer treatment duration of cyclosporine [34]. Interestingly, reactivation or reverse seroconversion has been reported 1–3 years after bone marrow transplantation. Hematopoietic stem cell recipients with a history of HBV exposure (HBsAg+ or HBsAg−) are therefore recommended to receive anti-HBV prophylaxis due to the risk of reactivation and associated morbidity and may require long-term prophylaxis.

#### 4.2.3. Other Immunotherapy: Checkpoint Inhibitors, Tyrosine Kinase Inhibitors, and mTOR Inhibitors (Everolimus)

Checkpoint inhibitors have revolutionized the treatment of many malignancies. A recent meta-analysis showed that patients with positive HBsAg had a pooled reactivation rate of ~11% without prophylaxis, with 1% developing reactivation-associated hepatitis [18]. In individuals with negative HBsAg and positive anti-HBc, 0.2% of patients developed HBV reactivation. As such, anti-HBV prophylaxis may be recommended in patients with positive HBsAg, though it is unlikely to be necessary in patients with negative HBsAg and positive anti-HBc.

Tyrosine kinase inhibitors have also had an increasing role in many oncologic regimens. Though available data are limited, the pooled rate of HBV reactivation was 11% in one meta-analysis of patients with positive HBsAg; conversely, no cases of reactivation in HBsAg− patients were reported. More recently, mTOR inhibitors have been used in some oncology regimens, though evidence is limited regarding their risk of HBV reactivation. Liver failure secondary to hepatitis B was described in one case study, [35] and another study found a 15% reactivation rate in patients with chronic hepatitis B, all of whom developed clinical hepatitis [36]. However, the risk in patients with negative HBsAg/positive anti-HBc is unknown.

Taken together, the available evidence suggests that antiviral therapy may be a reasonable consideration in HBsAg+ patients receiving treatment with these therapies, but the potential benefit in HBsAg− patients is less clear.

#### 4.2.4. Chemotherapy: Alkylating Agents (Cyclophosphamide and Etoposide) and Anthracyclines

HBV reactivation was first described in the context of chemotherapy, though the risk varies depending on the type of chemotherapy used. Alkylating agents such as cyclophosphamide or etoposide were associated with a 13% risk of HBV reactivation in one study in patients with positive HBsAg; interestingly, all of these patients developed clinical hepatitis [37]. Conversely, HBV reactivation does not seem to be a notable concern in HBsAg− patients, with no reported cases in one meta-analysis of available studies [18].

Anthracycline derivatives (doxorubicin and epirubicin) have also been associated with a high risk of reactivation in individuals with positive HBsAg; this may be particularly relevant in individuals undergoing TACE for hepatocellular carcinoma who are likely already at a higher baseline risk of reactivation. One study of patients receiving anthracyclines for breast cancer found that 20% of patients with positive HBsAg experienced HBV-reactivation-related hepatitis, with associations between hepatitis and the presence of older age and underlying liver disease (fatty liver or cirrhosis) on imaging [38]. This risk appears to be moderate, though lower, in individuals with negative HBsAg and positive anti-HBc, though the exact risk has not been defined [20].

#### 4.2.5. CAR T-Cell Immunotherapy

CAR T-cell immunotherapy is a relatively new treatment modality that uses genetically modified T cells to provide directed anti-tumor therapy and is often used in the context of hematologic malignancy. All available studies of patients with positive HBsAg receiving CAR T-cell immunotherapy involved patients taking antiviral therapy for HBV. Interestingly, this treatment has been associated with HBV reactivation in 11% of cases despite anti-HBV prophylaxis, including one case of HBV-reactivation-associated hepatitis [18]. Studies of HBsAg− patients were also undertaken. HBV reactivation was observed in 3% of patients with negative HBsAg/positive anti-HBC who did not receive anti-HBV prophylaxis (compared to 0% of patients receiving therapy), with one reported case of HBV-reactivation related hepatic failure and death. Overall, patients receiving CAR T-cell immunotherapy appear to be at a high risk of HBV reactivation and seem to benefit from anti-HBV prophylaxis. Some data may suggest that these patients may also benefit from prolonged prophylaxis after the completion of therapy, though evidence is limited to delineate the optimum duration.

### 4.3. Other Immunosuppressive Agents

#### 4.3.1. Glucocorticoids

Corticosteroids are used in a wide variety of malignant, autoimmune, and inflammatory conditions and are often used in combination with other therapies. Typically, high-dose systemic glucocorticoid use is defined as the use of a prednisone equivalent of ≥20 mg/day for at least four weeks. The dose and duration of corticosteroid use likely influence the risk of HBV reactivation. High-dose systemic glucocorticoid use has historically been classified as having a high risk of HBV reactivation in patients with positive HBsAg (an 11% pooled risk in one meta-analysis), while the risk is lower in patients with negative HBsAg/positive anti-HBc (3%) [18]. However, another study of HBsAg− patients suggested that individuals receiving a time-weighted average prednisone dose of ≥20 mg/day are at high risk of reactivation (particularly those anticipated to be on high-dose steroids for a prolonged period of time) and should be considered for anti-HBV prophylaxis [39]. Unsurprisingly, combining chemotherapy agents with steroids significantly increases the risk of HBV reactivation (26–72% compared to 13–26% without steroids) [9]. There also appears to be some risk of HBV reactivation in patients with positive HBsAg with the use of inhaled or low-dose systemic corticosteroids, though this has not been clearly delineated [9].

In recent years, the role of anti-HBV therapy in patients receiving steroids and other immunosuppressive therapy for COVID-19 (i.e., dexamethasone +/− tocilizumab or baricitinib) has been questioned. One study found that both HBsAg+ and HBsAg− patients did not appear to experience reactivation, but many were on anti-HBV therapy already [40] as the doses of dexamethasone used during COVID-19 would be associated with a high risk of reactivation in patients with positive HBsAg.

Overall, patients with positive HBsAg receiving high-dose steroids or steroids in combination with other immunosuppression likely benefit from anti-HBV prophylaxis due to their high risk of reactivation, while patients with negative HBsAg and positive anti-HBc should be evaluated on a case-by-case basis but may also benefit from antiviral prophylaxis.

#### 4.3.2. Antiproliferative Agents (Methotrexate)

Antiproliferative agents such as methotrexate are widely used in many rheumatologic conditions. A recent meta-analysis of available evidence found an 18% rate of HBV recurrence in patients with positive HBsAg without prophylaxis, while patients with negative HBsAg and positive anti-HBc did not experience any HBV reactivation [18]. However, this study represented low-quality evidence; antiproliferative agents such as methotrexate and azathioprine are widely viewed as having a low risk of HBV reactivation, and prophylaxis is not generally recommended [18,20].

#### 4.3.3. Calcineurin Inhibitors

Calcineurin inhibitors (CNIs) are frequently utilized in the context of solid organ transplant, often in combination with glucocorticoids. When used on their own, a recent meta-analysis found a 25% risk of HBV reactivation in patients with chronic hepatitis B, as well as a 10% risk of reactivation in HBsAg− patients, though the overall quality of evidence was low [18]. Interestingly, one study of CNI use in patients with negative HBsAg and positive anti-HBc reported a 2% development of severe liver decompensation and death in patients with HBV reactivation; notably, however, this study included hematopoietic stem cell transplant recipients who are already at a high risk of severe outcomes [34]. Current AASLD guidelines recommend solid-organ transplant recipients with positive HBsAg receive lifelong anti-HBV therapy starting at the time of transplant surgery; though the evidence is less clear in patients with negative HBsAg and positive anti-HBc; a limited period of anti-HBV therapy is reasonable [10].

#### 4.3.4. Cytokine Inhibitors (Tocilizumab, Ustekinumab, Secukinumab, and Siltuximab)

Cytokine inhibitors represent an alternative immunosuppressive regimen to anti-TNF agents in a variety of rheumatologic and autoimmune conditions, with many viewed as having more favorable side effect profiles. In patients with positive HBsAg, one meta-analysis found a pooled HBV reactivation rate of 36% without anti-HBV prophylaxis, though no episodes of hepatic decompensation or death were noted [18]. Other studies have noted a risk of HBV reactivation between 9 and 63% [21]. In patients with negative HBsAg and positive anti-HBC, the rate of HBV reactivation was 3%; another meta-analysis showed a pooled reactivation rate of 2–4% [41]. On their own, these therapies warrant consideration for prophylaxis; it is also important to note that the initiation of these agents is often accompanied by concurrent use of corticosteroids (e.g., in the case of a flare of an autoimmune disease), further increasing the risk of reactivation.

#### 4.3.5. Anti-TNF Agents (Infliximab, Adalimumab, Certolizumab, and Etanercept)

TNF-alpha inhibitors are frequently used in the treatment of inflammatory bowel disease, as well as other rheumatologic diseases. Anti-TNFs are known to have high risk of HBV reactivation in patients with chronic hepatitis B, suggesting a strong role for prophylaxis [20]. However, in HBsAg− patients, the role of prophylaxis is less clear—a recent meta-analysis categorized the pooled risk of reactivation as 1% in this group, with less than 0.1% going on to develop HBV-reactivation-associated hepatitis [18]; other studies have suggested that the risk in this group is low. There is also a different risk of reactivation associated with higher-potency agents in patients with positive HBsAg (e.g., infliximab, 12–39%) compared to lower potency agents (e.g., etanercept, 1–5%) [20]. Overall, most experts agree that prophylaxis is warranted in patients with positive HBsAg, whereas those with negative HBsAg and positive anti-HBc may be evaluated on a case-by-case basis.

#### 4.3.6. Janus Kinase (JAK) Inhibitors (Baricitinib and Tofacitinib)

JAK inhibitors have been utilized in rheumatologic conditions and, more recently, in inflammatory bowel disease. Data investigating the impact of JAK inhibitors on positive HBsAg are limited—one small retrospective cohort study showed that to 50% of patients with positive HBsAg developed HBV reactivation when treated with tofacitinib, while those who were also given prophylaxis did not [42]. Another study of HBsAg− patients saw that 14% of patients developed HBV reactivation, suggesting that these patients may also benefit from anti-HBV prophylaxis [43].

#### 4.3.7. T-Cell-Depleting Agents (Abatacept and Alemtuzumab)

T-cell depleting agents are utilized in many rheumatologic conditions. One meta-analysis estimated the risk of reactivation in patients with positive HBsAg at 9.5%, with no reported cases of reactivation in HBsAg− patients, suggesting a far lower risk of reactivation compared to B-cell-depleting agents (such as rituximab) [18]. However, data in this patient population are limited and warrant further investigation.

### 4.4. Other Immunosuppressed States

#### HIV Co-Infection

Though the term HBV reactivation typically refers to the consequence of starting an immunosuppressive medication, patients who are coinfected with HBV and HIV represent an important subset of patients in whom HBV flares may also occur. This can happen in the context of immune reconstitution or when antiretroviral therapies (ARVTs) with HBV activity are discontinued in the absence of HBsAg seroconversion. Current guidelines recommend that when ARVT regimens are altered, drugs that are active against HBV should not be discontinued without the addition of another drug with HBV activity. HBV treatment should also be continued indefinitely in this population [10].

## 5. Discussion

### 5.1. An Approach to Immunosuppression in Patients with HBV

**Figure 3:**
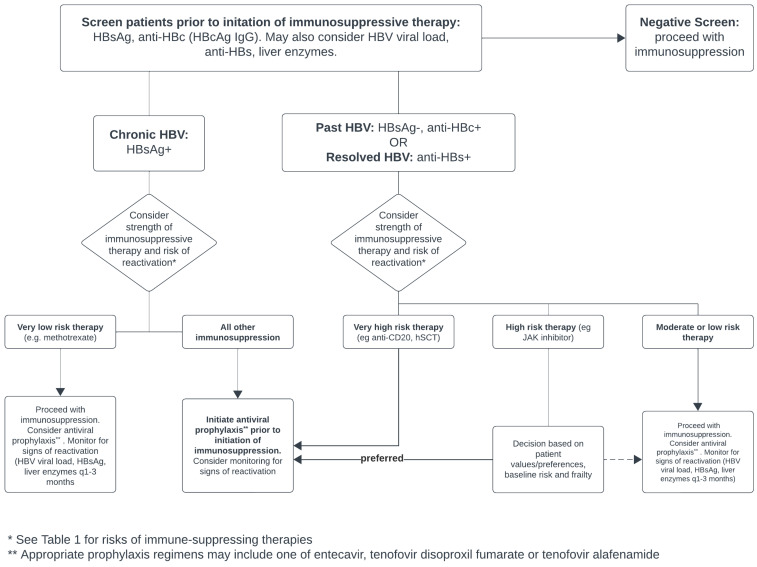
Proposed algorithm for screening and managing patients with HBV receiving immunosuppression. Adapted from [10,18,21].

Prior to the initiation of any immunosuppressive or immunomodulatory therapy (and particularly therapies at a moderate or high risk of HBV reactivation), most major societies recommend universally screening patients for HBV, regardless of local HBV prevalence [10,18]. Figure 3 illustrates a proposed algorithm for this approach.

#### 5.1.1. Screening

Routine screening should include, at minimum, serum HBsAg and anti-HBc (total or IgG) so that patients may be classified into one of the two main risk categories discussed above. The role of anti-HBs is less clear. The CDC recommends a triple-panel screening (HBsAg, anti-HBc, and anti-HBs) for all high-risk individuals [44], and a recent systematic review suggested that higher anti-HBs titers (>100 IU/L) may be protective against HBV reactivation, while low or negative titers may be associated with reverse seroconversion [45]. Similarly, a meta-analysis of patients with hematologic malignancies receiving chemotherapy found patients with resolved HBV (positive anti-HBs) had a decreased risk of reactivation compared to those who had negative anti-HBs [17]. However, current data are insufficient to recommend a definitive role for anti-HBs, particularly since the burden of antiviral prophylaxis is relatively low compared to the potential risks of HBV reactivation. As such, routine testing of anti-HBs may not be necessary at this time and may be left to the provider’s discretion. Clinicians may also wish to obtain baseline liver enzymes and function testing prior to beginning therapy. For patients receiving a form of therapy with a very low risk of HBV reactivation, screening may not be required.

#### 5.1.2. Antiviral Prophylaxis: Choice of Agent and Duration of Therapy

The use of anti-HBV prophylaxis in at-risk patients has been shown to reduce both rates of reactivation and associated morbidity and mortality. Current guidelines recommend the use of prophylactic first-line nucleot(s)ide analogues (NAs), such as entecavir, tenofovir disoproxil fumarate (TDF), or tenofovir alafenamide (TAF), due to their high resistance barrier and potency [10,30,46]. Pegylated interferon is not recommended. In patients with chronic hepatitis B (HBsAg positive), most studies recommend the initiation of NAs 7 days before the initiation of immunosuppressive therapy and continuation for at least 6–12 months after the discontinuation of therapy [10]. A longer duration of therapy may be warranted in patients receiving anti-CD20 therapy, as well as those with chronic immunosuppression, though evidence on the optimum length of therapy in these cases is limited.

#### 5.1.3. Prophylaxis and Suggested Monitoring

Current guidelines suggest that any patient with chronic hepatitis B should start anti-HBV prophylaxis prior to the initiation of immunosuppressive therapy due to the potential risks of reactivation. Similarly, HBsAg− patients starting therapy associated with a very high risk of reactivation (e.g., anti CD20 therapy or an HSCT transplant) should receive prophylaxis [10].

For patients with past or resolved HBV receiving other immunotherapies, the role of prophylaxis is less clear. Papatheodoridis et al. proposed that for patients receiving treatments with an intermediate risk of reactivation, clinicians may decide between anti-HBV prophylaxis and closely monitoring ALT and HBV DNA with the on-demand initiation of anti-HBV therapy when reactivation is detected [18]. EASL also suggests monitoring HBsAg due to the high risks of acute hepatitis in patients who undergo HBsAg reverse seroconversion [30]. Therefore, for patients in whom NA prophylaxis is not started (low or intermediate risk), HBV DNA, HBsAg, and ALT levels should be obtained every 1–3 months. In the event of HBsAg reverse seroconversion, NA therapy should be started promptly due to high risks of acute hepatitis.

#### 5.1.4. Treatment of HBV Reactivation

HBV reactivation should be managed promptly with NAs (TDF, TAF, or entecavir); the treatment of HBV reactivation with interferon is not recommended by major society guidelines. There are limited data on the efficacy of antiviral treatment at preventing morbidity and mortality related to HBV reactivation, though one review estimated the survival rate of patients who progress to liver failure at <20% [47,48]. Similarly, data regarding liver transplant in the case of acute or acute on chronic liver failure in this population are limited to case reports [49,50], possibly because members of the at-risk population often have other contraindications to transplant, such as active malignancy. In patients with suspected hepatitis related to HBV reactivation, other causes of elevated liver enzymes should be ruled out. The modification or interruption of immunosuppression remains controversial and should be considered on a case-by-case basis in consultation with other members of the patient’s circle of care (e.g., oncologists, rheumatologists), with a discussion of the risks and benefits of stopping immunosuppression. Further studies are required to better understand the optimum thresholds for and timing of stopping immunosuppression in this context.

### 5.2. Future Directions

Predicting and managing HBV reactivation is a significant consideration in patients receiving immunosuppressive therapy. While the development of treatments that can completely eradicate HBV cccDNA from hepatocytes remains the ultimate goal, future studies should also seek to better understand the risk factors associated with the development of HBV reactivation and clinically relevant hepatitis, as well as the true rates of HBV reactivation, hepatitis, and mortality associated with various treatment modalities. Although patients with prior HBV exposure have, in general, been excluded from clinical trials, future trials should consider including these patients with close monitoring in order to allow for a better understanding of the risk of reactivation with novel therapies. Finally, studies delineating the optimum duration of antiviral prophylaxis after the completion of therapy (or in the case of chronic immunosuppressive agents, if these agents can safely be stopped) will allow clinicians to better streamline patient care.

## 6. Conclusions

Hepatitis B reactivation can affect individuals with chronic or past hepatitis B receiving immunosuppressive agents, and in some cases, may cause clinically important hepatitis or liver failure. Clinicians should attempt to understand patients’ baseline risk of HBV reactivation prior to initiating immunosuppressive therapies and initiate appropriate anti-HBV prophylaxis where appropriate. In patients who do not receive prophylaxis, HBV DNA levels, HBsAg and liver enzymes should be monitored to allow for the prompt recognition and treatment of HBV reactivation.

## Figures and Tables

**Table 1:** Risk of HBV reactivation based on immunosuppressive agent. Adapted from [10,18,20,21].

Risk of Reactivation	HBsAg Positive (Chronic HBV)	HbsAg Negative, anti-HBc Positive (Past HBV)
High (>10%)	Anti-CD20 * Hematopoietic stem cell transplant Immune checkpoint inhibitors Cytokine inhibitors Tyrosine kinase inhibitors CAR-T cell immunotherapy Corticosteroids (≥20 mg/day for ≥4 weeks) Alkylating agents Anti-proliferative agents Calcineurin inhibitors mTOR inhibitors Janus kinase (JAK) inhibitors	Janus Kinase (JAK) inhibitors
Moderate (1–10%)	T-cell-depleting agents Anti-TNF (without steroids) ^+^ Anti-rejection (without steroids)	Anti-CD20 **^,+^ Cytokine inhibitors CAR-T cell immunotherapy Corticosteroids (≥20 mg/day for ≥4 weeks) Calcineurin inhibitors Hematopoietic stem cell transplant ^+^
Low (<1%)	Methotrexate Azathioprine	Anti-TNF agents ^++^ Immune checkpoint inhibitors Tyrosine kinase inhibitors T-cell-depleting agents Alkylating agents
Rare		Methotrexate Azathioprine Cytotoxic chemotherapy without steroids
Unknown		mTOR inhibitors

* in some cases, classified as very high (>20%). ** treatment recommended by guidelines. ^+^ classified as high-risk by the Asian Pacific Association for the Study of the Liver(APASL) [21]. ^++^ classified as moderate risk by the APASL [21].

## Data Availability

No new data were created or analyzed in this study. Data sharing is not applicable to this article.
